# Sustainable Approach of Functional Biomaterials–Tissue Engineering for Skin Burn Treatment: A Comprehensive Review

**DOI:** 10.3390/ph16050701

**Published:** 2023-05-05

**Authors:** Loai A. Elfawy, Chiew Yong Ng, Ibrahim N. Amirrah, Zawani Mazlan, Adzim Poh Yuen Wen, Nur Izzah Md Fadilah, Manira Maarof, Yogeswaran Lokanathan, Mh Busra Fauzi

**Affiliations:** 1 Centre for Tissue Engineering and Regenerative Medicine, Faculty of Medicine, University Kebangsaan Malaysia, Kuala Lumpur 56000, Malaysia; loailol62@gmail.com (L.A.E.); manira@ppukm.ukm.edu.my (M.M.); lyoges@ppukm.ukm.edu.my (Y.L.); 2 Department of Surgery, Hospital Canselor Tuanku Muhriz, Universiti Kebangsaan Malaysia, Kuala Lumpur 56000, Malaysia

**Keywords:** skin burn, sustainable biomaterials, multifunctional bioscaffolds, collagen, cellulose, chitosan, quercetin, tissue engineering

## Abstract

Burns are a widespread global public health traumatic injury affecting many people worldwide. Non-fatal burn injuries are a leading cause of morbidity, resulting in prolonged hospitalization, disfigurement, and disability, often with resulting stigma and rejection. The treatment of burns is aimed at controlling pain, removing dead tissue, preventing infection, reducing scarring risk, and tissue regeneration. Traditional burn wound treatment methods include the use of synthetic materials such as petroleum-based ointments and plastic films. However, these materials can be associated with negative environmental impacts and may not be biocompatible with the human body. Tissue engineering has emerged as a promising approach to treating burns, and sustainable biomaterials have been developed as an alternative treatment option. Green biomaterials such as collagen, cellulose, chitosan, and others are biocompatible, biodegradable, environment-friendly, and cost-effective, which reduces the environmental impact of their production and disposal. They are effective in promoting wound healing and reducing the risk of infection and have other benefits such as reducing inflammation and promoting angiogenesis. This comprehensive review focuses on the use of multifunctional green biomaterials that have the potential to revolutionize the way we treat skin burns, promoting faster and more efficient healing while minimizing scarring and tissue damage.

## 1. Introduction

Burn, a traumatic injury that leads to a huge disruption in skin structure and function, is a widespread public health problem. Through energy transfer from the causative agent to tissues, heat, cold, friction, electricity, radiation, and chemicals could induce a serious burn injury [1]. Many causative agents are associated with other physiological and pathophysiological responses. A superficial level-up to deep burn might happen after direct exposure to hot grease and liquids, fire, and steam. Another thermal burn type that can damage deep tissue is frostbite burn. Colliquative and coagulation necrosis might happen upon contacting with alkaline or acidic chemicals [2]. The deep tissues affected directly after exposure to electrical shock are greater than the superficial skin injury, which is correlated with the strength of the electric field [3]. According to WHO, there are approximately 180,000 deaths per year due to burns. The highest percentage goes to low- and middle-income countries. In India, over one million moderate to severe burns cases are admitted to hospitals annually. In addition, 17% to 18% of children have temporary and permanent disabilities after burning in Egypt, Colombia, Pakistan, and Bangladesh. The second cause of common injury in Nepal, with 5% of disabilities, is burning. In 2008, around 40,000 patients were hospitalized with different degrees of burn injuries in the United States of America (2018).

Tissue engineering and regenerative medicine (TERM) is one of the most rapidly evolving multidisciplinary fields, which develops several principles for repairing, regrowing, or even replacing diseased or damaged tissues and organs [4]. It combines basic sciences such as material science, biochemistry, biomechanics, polymer chemistry, cell biology, and nanotechnology with applied medical and engineering sciences [5]. In the last few decades, human beings have been dreaming of repairing and restoring the functions of damaged tissues and organs, but now, huge progress has been accomplished. Despite the tremendous progress, TERM is still in its infancy. Bioengineers are trying to extract and innovate new biomaterials that could be a base of engineered replacement tissues to facilitate burn healing. These biomaterials are designed to interact with the biological system of the skin to be used as an alternative extracellular matrix for replacing or augmenting natural skin tissue and to provide support structures or scaffolds for regenerative medicine applications such as implants, drug delivery devices, and tissue engineering scaffolds. Biomaterials must be biocompatible to minimize the risk of adverse effects and immune reactions toward the fabricated scaffolds [6]. They include natural and synthetic polymers such as collagen and silicones, metals and ceramics such as titanium alloys and zirconia, and nanomaterials such as carbon nanotubes.

Non-ecological biomaterials have been replaced by green biomaterials or, in other words, “sustainable biomaterials” that open new opportunities in their therapeutic applications in 3D bioprinting, tissue engineering, drug delivery, and the fabrication of different types of scaffolds. The interaction and coexistence of these natural biomaterials inside human bodies could be safe if they achieved the minimum requirements needed. They must be non-toxic, biodegradable, and biocompatible, especially for these green biomaterials that are used to be a scaffold for cells to grow on it. Many aspects have been used to describe the responsiveness of green biomaterials [7]. Most the sustainable biomaterials are saccharide repeat units in nature [8]. However, some of them are body-like components due to the presence of amino acid repeat units that enhance biodegradability and biocompatibility for biomedical uses [9].

Naturally extracted biomaterials are commonly obtained from algae, plant, and animal sources [10] either by enzymatic processes or the fermentation of microorganisms [11]. Animal waste, mainly produced by the meat, leather, and poultry industries, is abundant and includes non-edible parts such as bones, feathers, tendons, and skins. These wastes contain pathogens and require proper treatment. Common treatments include burning them or composting them with manure, although both methods have drawbacks, including high energy consumption, carbon dioxide emissions, an unpleasant H2S smell during composting, and others. Current research has focused on finding sustainable applications for animal waste, including the extraction of biomaterials (collagen, gelatine, chitosan, and elastin), the generation of biogas, and the production of commercial crops and biodiesel [12]. In addition to animal waste, human skin also contains abundant bio-materials that can be used for sustainable applications such as the extraction of human collagen. For instance, the human skin has a redundant property, which means that it can be removed during surgery or cosmetic procedures and still be functional. This redundant skin can be repurposed as a valuable bioscaffold material for tissue engineering and wound healing applications. Researchers are exploring various methods for treating and processing redundant human skin to create bioscaffolds that mimic the natural extracellular matrix, providing a supportive environment for cell growth and tissue regeneration. This sustainable reuse of human skin can not only reduce waste but also offer an alternative to traditional synthetic materials, ultimately improving patient outcomes.

Sustainable biomaterials are classified chemically into two main groups: polysaccharides in nature such as chitosan, alginate, carrageenan, cellulose, pectin, agarose, and hyaluronic acid [13] and protein such as collagen, gelatin, silk, keratin, and fibrin. Several synthetic biomaterials have also been used in skin burn treatment for several purposes. Researchers are developing synthetic biodegradable polymers that can be used as scaffolds for skin tissue repair. These attempts include using antimicrobial agents, antioxidants, growth factors, cytokines, and other supplements incorporated inside these biomaterials to be fabricated as multifunctional scaffolds to accelerate burn healing by promoting endogenous cell migration and proliferation. It opens a new era for exploiting the treasures of mother nature to adapt them in the innovation of new green biomaterials to be used not only in tissue engineering but also in drug delivery systems, drug targeting approaches, allo- or auto-transplantation, and molecular medicine. This review presents a comprehensive update on using sustainable biomaterials in skin burn treatment, with special emphasis on the mechanisms underlying burn wound healing and recent advancements in burn wound care under the umbrella of multifunction biomaterials.

## 2. Burn Pathophysiology

**Figure 1:**
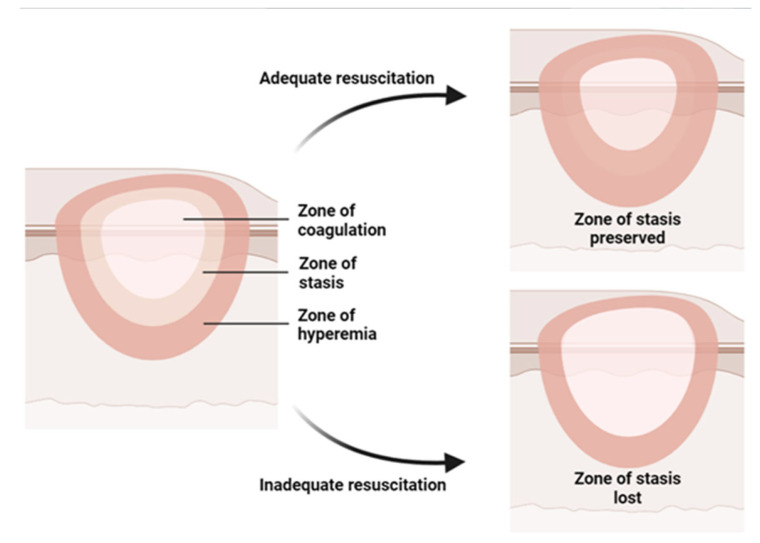
Illustration of the three concentric zones of thermal burn that can be recognized in and around the burned skin: the zone of coagulation is in the center, which is the area closest to the source of the burn and experiences the most intense damage; the zone of stasis, which is the area surrounding the zone of coagulation, where the damage is less severe but still significant; the zone of hyperemia, which is the outermost area surrounding the burn injury, where the tissues are minimally affected.

Burn injury involves both local and systemic responses. Regarding local reaction, a burn can be described by Jackson’s (1953) three concentric zones on the first day of injury: central coagulation, intermediate stasis, and outer hyperthermia zones (Figure 1) [14]. There are three zones of injury in a burn: the central zone of coagulation, the intermediate zone of stasis, and the outer zone. The central zone appears white due to irreversible tissue damage, while the intermediate zone has compromised blood supply and reversible tissue injury with possible petechial hemorrhages. Prompt treatment to increase tissue perfusion is necessary to prevent progression to the central zone [15]. The outer zone sustains the least damage and can heal within a week, but the healing time varies depending on the depth of the wound, the site of burning, and the patient [15].

Burns over 30% of the total body surface area (TBSA) cause systemic response and shock. Pathological changes in cardiovascular, renal, respiratory, and gastrointestinal systems and metabolism are observed in burn shock resulting from direct tissue injury, hypovolemia, and elevated levels of cytokines and inflammatory mediators in the systemic circulation [16]. Severe burns cause increased vascular permeability and vasodilation, leading to fluid leakage from capillaries into interstitial spaces, causing edema in both burned and unburned areas. This loss of fluid leads to poor circulation, tissue ischemia, and organ dysfunction. Burn resuscitation is necessary to replace lost fluids and maintain organ perfusion [17].

Burn injuries activate a two-phase proinflammatory and anti-inflammatory response [18,19]. Tissue damage from burns releases endogenous damage-associated molecular patterns (DAMPs), leading to a vascular leak, an immune response, and metabolic changes. The transcriptional activator NF-κB is activated after severe burn injury, inducing inflammatory mediators such as interleukins 1, 6, and 8 and TNF, which cause systemic effects [20]. Burn injuries result in an immediate inflammatory reaction that aids in tissue repair, similar to other injuries [18,19,20]. Systemic inflammatory response syndrome, characterized by uncontrolled cytokine release and compromised adaptive immune response, increases the risk of infection in severely burned and injured patients [21]. Hypermetabolism after burn is reviewed extensively in other reviews [22,23]. The profound and prolonged hypermetabolism driven by systemic inflammation, stress hormone release, and reactive oxygen species (ROS) formation in burn injury leads to multi-organ failure and septic complications [24,25,26].

### 2.1. Wound Healing Mechanism

**Figure 2:**
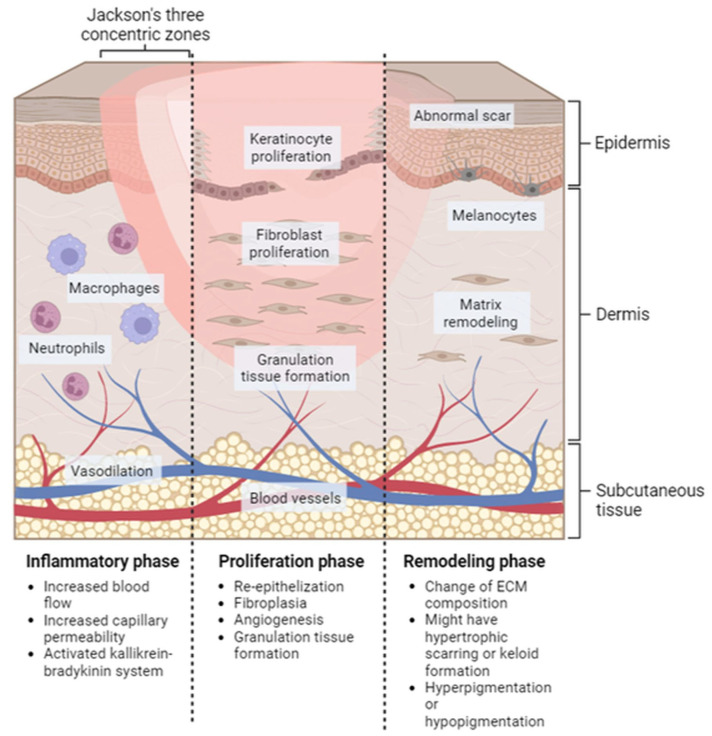
Localized natural phases of the wound healing process for burn injury. Inflammatory phase: this stage starts immediately after the injury occurs and lasts for approximately 3–5 days. Proliferation phase: this stage typically lasts from days 5 to 21 after the injury. Remodeling phase: this can last for several months or even years after the injury.

Wound healing is a systematic process that involves multiple cell types, the extracellular matrix, and mediators. There are four overlapping and time-dependent phases of natural wound healing: (i) hemostasis; (ii) inflammation; (iii) proliferation; and (iv) remodeling. Due to the specific nature of burns, burn healing differs from natural wound healing. When it comes to thermal burns, the initial inflammatory response may be more pronounced due to the extent of tissue damage and the release of cytokines and other inflammatory mediators. However, the subsequent phases of wound healing, including proliferation and remodeling, may also be impacted by the extent of the injury and other factors such as infection and underlying medical conditions. The burn wound healing depends on the depth and severity of the burns, according to the involvement of the epidermis, dermis, or deeper tissues. The repair process of extensive burn injuries (involving skin and deeper tissues) is more complex and slower, leading to increased scar formation (Figure 2) [27].

#### 2.1.1. Hemostasis

Hemostasis is the shortest phase in wound healing and occurs immediately after injury. It involves vasoconstriction followed by vasodilation to stop bleeding. Platelets, macrophages, keratinocytes, and fibroblasts release clotting and growth factors such as platelet-derived growth factor (PDGF), epidermal growth factor (EGF), and transforming growth factor-β (TGFβ) to form a scaffold for infiltration cells that aid in subsequent healing phases [18].

In burn wounds [18], the hemostasis phase of wound healing is not commonly considered [28]. This is because there is no bleeding in the burn wound, and there is increasing capillary permeability, which causes fluid leakage from capillaries to interstitial spaces, leading to plasma loss. If this plasma loss is not replenished, it can cause hypovolemic shock, which affects myocardial contractility and vasoconstriction in peripheral and splanchnic circulation [29].

#### 2.1.2. Inflammation

Inflammation occurs within 24 h and lasts for weeks to months from the injury. It helps clean pathogens and foreign materials from the wound, degrades necrotic tissue, and initiates signaling cascades that are necessary for wound healing. Local histamine release increases blood flow and vascular permeability during the early inflammatory phase, allowing inflammatory cells to migrate to the injury site. Neutrophils and macrophages are recruited by the complement cascade and the release of inflammatory cytokines and mediators, and they help to phagocytose bacteria, foreign materials, and damaged tissue [30]. In the late inflammatory phase, macrophages recruit and activate lymphocytes to the injury site. Lymphocytes secrete lymphokines that aid in the wound healing process. The role of lymphocytes is not fully understood, but the different level of T-cells showed different effects in wound healing [31,32]

During the inflammatory phase of thermal wound healing, histamine released from mast cells causes vasodilation and increased capillary permeability, leading to a loss of intravascular fluid and potential hypovolemic shock. Severe burn injury can activate the kallikrein–bradykinin system, leading to the production of bradykinin, which causes vasodilation, increased permeability, smooth muscle contraction, and pain [33]. Other potent vasoactive mediators, including prostaglandins, prostacyclins, and thromboxanes, are produced from arachidonic cascade after burn injury, causing the exacerbation of edema formation, local tissue ischemia, and a predisposing condition for thrombus formation [34]. Studies showed that the immune response of thermal burn injuries is dominated by innate immune cells [35]. The cellular immune response is vital in phagocytosis and kills bacteria. An immunocompromised patient with severe burn injury has increased susceptibility to infection.

#### 2.1.3. Proliferation

Proliferation occurs when the inflammation subsides, begins on the fourth day after wounding, and extends 14 days thereafter. This phase involves re-epithelization, fibroplasia, angiogenesis, and granulation tissue formation [36]. Keratinocytes and epidermal stem cells from hair follicles and apocrine glands assist in re-epithelization. Keratinocytes from the basal layer of wound edges are activated after injury and undergo partial epithelial–mesenchymal transition to become more invasive and migratory. Re-epithelization starts with migrating keratinocytes from the basal layer of wound edges and the differentiated keratinocytes from epidermal stem cells. Matrix metalloproteinases (MMPs) (MMP-1 and MMP-9) and other proteases such as plasmin are vital for keratinocyte migration [36]. Once the keratinocytes from opposing edges meet, they stop migrating and reform the basement membrane. Keratinocytes then undergo terminal differentiation to stratify and generate a stratified epidermis. The reconstitution of the dermis is characterized by fibroplasia, granulation tissue formation, and angiogenesis. Fibroblasts in the wound edges proliferate, migrate into the provisional wound clot matrix, and begin extracellular matrix ECM production. Myofibroblasts, differentiated phenotypes from fibroblasts, are stimulated to participate in wound contraction. Angiogenesis occurs to supply oxygen and nutrients to highly proliferative healing tissue. Microvascular endothelial cells react to various factors, including hypoxia-inducible factors (HIFs), VEGF, FGFs, and hepatocyte growth factor (HGF), and proliferate and migrate into the wound bed to form new blood vessels. Macrophages produce proteases and other chemotactic factors, such as TNF-α, VEGF, and TGF-β, which aid microvascular endothelial cells in angiogenesis.

The proliferation process of burn wound healing depends on the severity of burns [37]. In superficial (first-degree) burns, keratinocytes proliferate and migrate to restore the normal layer of the epidermis, and the wound will recover within two weeks with minimal scarring. In deeper burn wounds, re-epithelization occurs, and the healing begins from skin appendages. Then, fibroplasia and angiogenesis occur to restore and reconstruct dermal tissue [37].

#### 2.1.4. Remodeling/Maturation

Remodeling is the last and longest phase of wound healing. Remodeling can last for months to years, and it results in the final appearance of the wound after healing. The granulation tissue experiences a change in ECM composition in this phase, where the granulation tissue with predominant collagen type III is replaced by collagen type I, which has a higher tensile strength of the forming scar [38]. Fibroblasts and myofibroblasts play a major role in ECM remodeling by secreting ECM, MMPs, and tissue inhibitors of metalloproteinases. Various regulatory mechanisms tightly control the degradation of old matrix and the synthesis of the new matrix to accomplish successful and normal wound healing. Hypertrophic scarring or keloid formation happen when excessive fibrosis occurs during wound remodeling. The tensile strength can never be regained, and the wound scar tissue (healed tissue) can only achieve 80% of the pre-wounding tensile strength. The distribution of mature elastin fibers is obviously seen after months from the injury [39]. Declined angiogenesis results in decreased metabolic activity at the wound site. Lastly, a fully matured scar with a high tensile strength is produced.

This remodeling phase is almost identical to every wound, including burn wounds. The depth and severity of the burns determine how each of these steps will play out. Melanocytes would overreact with the burns and result in hyperpigmentation or cause hypopigmentation when the lower levels and basal layer of the epidermis are destructed [40,41]. The delayed healing in severe burn injuries and excessive inflammation may contribute to excessive pigmentation and hyperpigmentation. In severe and deeper burns, prolonged remodeling also increases the probability of hypertrophic scarring and contractures.

## 3. Biomaterials

### 3.1. Definition

Dr. Jonathan Cohen provided one of the earliest definitions of “biomaterial”, which is “any material used as implant”, in 1967 [42]. the advancement of tissue engineering technology has led to a redefinition of the term "biomaterial" from referring to "an inactive substance", "substances that are both active and inactive", and "a material lacking viability" to now signify "an enhancement of quality of life" [43]. A new definition of “biomaterial” was published in 2019 and is now defined as “a material designed to take a form that can direct, through interactions with living systems, the course of any therapeutic or diagnostic procedure” [44]. All the definitions above surround a keyword, which is “biocompatible”. It has been shown that natural biomaterials have several advantages over synthetic biomaterials. They are remodeling, biodegradability, and biocompatibility.

### 3.2. Application of Biomaterials

Biomaterials were used to solve body issues by Neanderthals 40,000 years ago. In the 19th century, biomaterial science and technology were promoted in line with the emergence of the manufacture of prostheses and implants. Biomaterial science is a multidisciplinary field, and it is supported by biology and biomechanics and material science [45]. To date, biomaterials are used in a broad range of applications, including medical implants, protheses, surgical sutures, supporting scaffolds, and molecular probes and nanoparticles that aid in imaging and therapy [46]. One of the most important applications for biomaterials is in the field of small-diameter vascular graft (SDVG) engineering. It is a field of research that focuses on developing artificial blood vessels with diameters of less than 6 mm for use in vascular surgeries [47,48]. The goal of SDVG engineering is to create a graft that is biocompatible, non-thrombogenic, and durable and can integrate with the surrounding tissue to promote natural healing and enhance the blood flow to the target tissue [49].

### 3.3. Modern Approaches

Biomaterials have played a crucial role in the development of medical treatments, prosthetic devices, and tissue engineering. Traditionally, biomaterials have been derived from natural sources such as plants and animals. However, recent advancements in biomaterials research have led to the emergence of novel approaches such as the decellularized matrix, stem cells, and synthetic biomaterials.

#### 3.3.1. Decellularized Matrix

The decellularized matrix involves the removal of cellular components from natural tissues, leaving behind a scaffold that can be repopulated with cells to regenerate new tissue [50]. Since Poel’s initial investigation in 1948, there has been significant research conducted on decellularized extracellular matrices (dECMs) derived from organs and tissues [51]. These dECMs possess characteristics of an ideal tissue scaffold, such as a distinct tissue-specific structure, intricate vascular network, and composition, making them suitable for applications in both in vitro and in vivo regenerative medicine [52]. This approach has several advantages, including the preservation of the extracellular matrix and its associated signaling molecules, which can help guide cell behavior and promote tissue regeneration [53]. They create an environment that promotes the growth and differentiation of stem cells and have properties that can be enhanced by combining various natural tissue-engineering materials. The decellularized matrix has been used in a variety of applications, including cardiac tissue engineering, bone regeneration, and skin replacement [54,55,56,57]. There are many disadvantages this approach faces in repairing different organs and tissues in humans. Clinical studies have reported non-infectious edema, severe pain, scar formation, fibrotic scar, poor biomechanical properties, and inflammatory responses [58,59,60,61,62,63,64,65]. Nevertheless, further research is needed to evaluate these materials in vivo through animal experiments while also taking into account the differences between animal models and humans. In summary, refunctionalizing dECMs appears to be a promising strategy for tissue engineering and regenerative medicine.

#### 3.3.2. Stem Cells

Stem cells have shown great potential in regenerative medicine for the treatment of skin burns. Stem cells are currently one of the most widely studied topics in modern medicine and biological sciences due to their unique properties, including the ability to differentiate into various cell types and their potential for unlimited self-renewal. Stem cells can be categorized based on their differentiation potential, such as totipotent, pluripotent, multipotent, or unipotent, and their source, such as embryonic stem cells derived from the blastocyst, adult stem cells isolated from mature organisms (also known as somatic stem cells), or stem cells obtained from the umbilical cord or placenta. One specific type of stem cells, known as induced pluripotent stem cells (iPSCs), can be generated from adult cells through the introduction of specific genes encoding transcription factors [66]. Mesenchymal stem cells (MSCs) play a crucial role in all stages of wound healing, and utilizing them in skin therapy can promote wound healing and reduce scarring. MSCs migrate to the site of skin injury, suppress inflammation, and enhance the proliferation and differentiation capacity of fibroblasts, epidermal cells, and endothelial cells. MSCs have been found to facilitate the regeneration of neurons. Essential secretory factors such as bFGF, nerve growth factor (NGF), and brain-derived neurotrophic factor (BDNF) play a significant role in promoting the regeneration of nerves [67,68,69].

Numerous studies have demonstrated the potential of MSCs in the treatment of burn injuries, with reports indicating that MSCs can enhance wound closure, promote angiogenesis, and reduce scarring [70,71,72,73]. Many studies have reported the role of MSCs in facilitating the burn healing by upregulating the expression of collagen 1 and integrin α2β1 [74]. It is also promoting burn healing through an immunosuppressive mechanism [75]. However, the underlying mechanisms by which MSCs facilitate burn healing are not yet fully understood. Additionally, the attachment of transplanted MSCs to wounds is limited, and the rate of engraftment into organs has been reported to be less than 3% in various injury models such as heart, kidney, liver, and pancreatic injuries [76,77,78,79]. As a result, more detailed studies are needed to improve the engraftment rate of MSCs in damaged skin. Two methods of MSC delivery have been developed: local delivery via various scaffolds embedding MSCs fabricated from natural or synthetic biomaterials and systemic delivery via intracardiac, intramuscular, or intraperitoneal injections or intravascular injection (either arterial or venous) [80,81]. Therefore, the further development of these two MSC injection methods is necessary to increase the rate of engraftment and engagement of MSCs in damaged skin.

### 3.4. Biomaterials in Burn Skin Healing

Skin grafting, a surgical procedure that involves skin transplantation, is the common treatment for extensive burn injuries. The donor can be the same individual (autograft), another person (allograft), from a different species (xenograft), or a deceased person (post-mortem allograft) [82]. There are four major categories of skin grafts based on the composition, which are full-thickness skin graft (FTSG), split-thickness skin graft (STSG), epidermal skin grafts (ESG), and composite graft. Skin grafting is time-consuming, as it involves a second surgical site [83]. Graft failure may happen as a result of inadequate recipient site excision, graft rejection, shear stress, or wound infection [18].

Applying biomaterials improves patients’ quality of life and survival rate by promoting tissue regeneration and preventing tissue deterioration [84]. For the treatment of wounds and burns, there are a plethora of options available, the majority being wound dressings. Occlusive wound dressings create a sealed wound environment to prevent infections and maintain moisture to accelerate wound healing, while non-occlusive dressings allow for the movement of air and eliminate moisture from the wound [85]. Wound dressing can be incorporated with biologics, materials, or any agent that is known to have antibacterial properties or other biologic properties for promoting the events in skin healing and regeneration. Examples of wound dressings include fiber mats and hydrogels.

Currently, skin tissue engineering is emerging to generate bioengineered skin substitutes for intensive burn injury. Skin substitutes consist of heterogenous groups of materials (can be cellular or acellular) that possess skin composition and function, provide wound coverage temporarily or permanently, and promote autologous regenerative healing, with a minimal inflammatory response [86,87]. These bioengineered skin substitutes are created using natural or synthetic polymers such as collagen, gelatine, chitosan, quercetin, hyaluronic acid natural polymers, polyethylene glycol (PEG), polylactic-co-glycolic acid (PLGA), and inter alia synthetic polymers. The three main types of skin substitutes are epidermal cover, dermal replacement, and dermo-epidermal replacement. These tissue-engineered skins provide skin barrier function, vasculature, elasticity, and support and secure attachment to the dermis, supporting the wound microenvironment for wound healing and skin regeneration [88]. Examples of current skin substitutes are listed in Table 1.

## 4. Protein-Based Biomaterials

**Figure 3:**
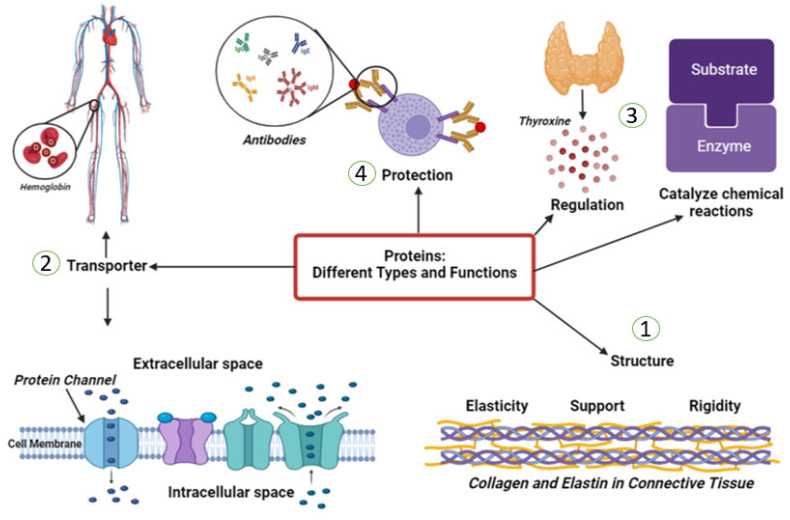
This figure describes some of the different types and functions of proteins in the human body. (1) Structural proteins provide support and shape to cells and tissues in the body such as collagen. (2) Transport proteins help to move molecules such as nutrients and oxygen throughout the body like hemoglobin. (3) Enzymes that catalyze, regulate, or speed up the chemical reactions in the body. (4) Antibodies are proteins that help to identify and neutralize foreign substances and protect the human body from them.

Approximately 50% of vertebrates’ tissue dry weight consists of proteins. They are the most abundant organic compounds in human bodies, and almost all biological processes depend on them. The major components of human skin are proteins, which provide a suitable microenvironment for keratinocytes and fibroblasts. Over the past 60 years, significant progress has been made in the understanding of the function and structure of proteins [112]. This has helped identify the molecular basis of the numerous tacit biological processes. Proteins have lots of types; each plays its own role in our bodies. It could be hormones that regulate many cellular activities, enzymes that catalyze plenty of chemical reactions in the human body, transporters that carry series of substances, and molecules in blood, such as hemoglobin, or the cell’s membrane. They act as defense weapons against any foreign bodies’ attack and are known as antibodies. Collagen is a highly resistant fiber in connective tissue. (Figure 3).

**Figure 4:**
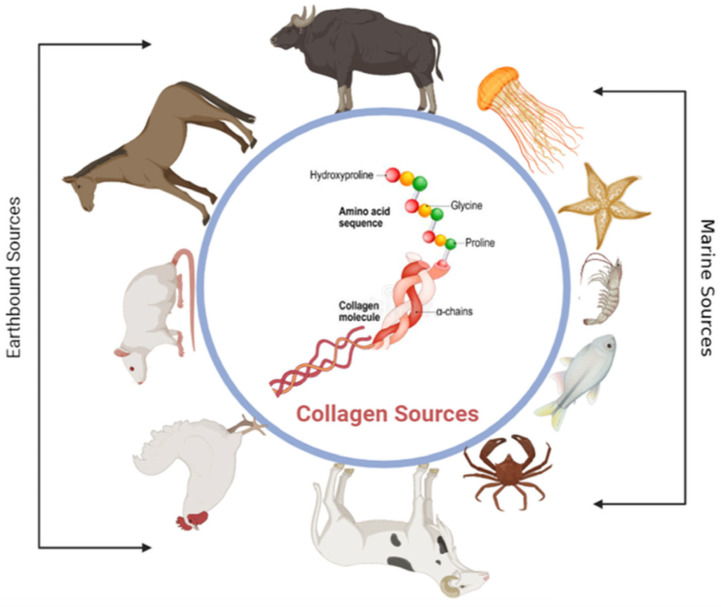
This figure shows different natural sources of collagen. The sources include earthbound sources such as bovine, horses, rat tail, chicken, and ovine and marine sources such as fishes, crabs, shrimp, etc.

In the animal kingdom, there are lots of sources of proteins to extract, which have been documented back to the early 1950s [113,114]. The following are only a few examples of different sources of proteins: bovine and ovine tendons, pigs’ skin, rat tails, chicken, horses, jellyfish, craps, and starfish (Figure 4) [115]. Collagen, gelatin, keratin silk, and fibrin are the most common proteins used to fabricate scaffolds in biomaterials applications. One of the biggest challenges investigators face with proteins is the extraction, isolation, and purification from the complex mixture of substances that composes living matter. This makes the development of new methodologies and technologies for separating proteins in a pure form an urgent demand. One successful method is chemically based for extracting collagen type I from ovine tendons, followed by lyophilization to obtain the proteins in fibers form [116]. It will help in understanding the structure and function of many proteins from different sources.

### 4.1. Collagen

Collagen is a main protein found throughout the body, and it plays several essential roles in maintaining the structural integrity and function of tissues. One of the most critical roles of collagen is in wound healing, as it is a key component of the body’s repair process following injury or damage to the skin or other tissues. It maintains the biological integrity of the ECM. A total of 30% of the total human body protein consists of different collagen types. They play various roles in cell adhesion and migration, scaffolding, and repair for human tissue [117]. A total of 28 types of collagen that have been discovered up until now have different molecular structures, with a specific function for each one of them [118].

**Figure 5:**
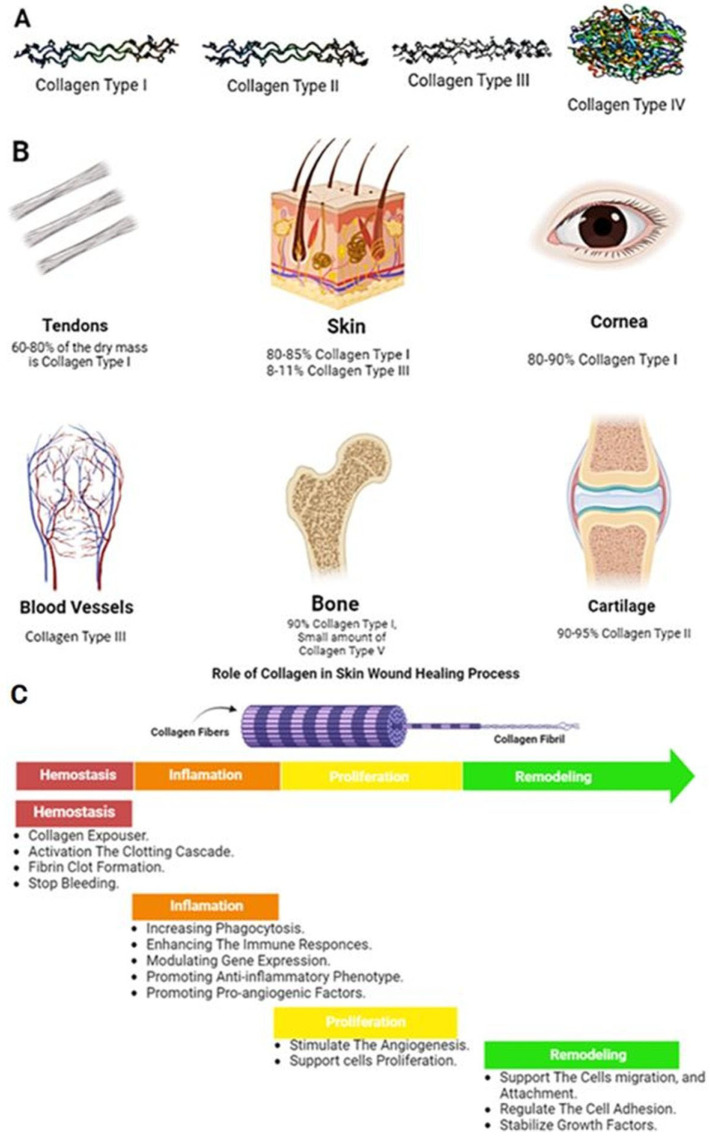
(**A**) Examples of different three-dimensional shapes of some types of collagen, (**B**) Approximate content of collagen type I in different tissues, (**C**) The role of collagen in the four phases of the wound healing process.

Collagen fibers are arranged in a specific pattern and provide structural support to tissues. In skin, collagen provides structural support and helps to maintain the skin’s elasticity. Collagen type I constitutes 80–85% of the dermal extracellular matrix, with 8–11% of collagen type III [119]. More than 90% of collagen type I and a small amount of type V are building the bone structure [120]. Tendons and ligaments are connective tissues that connect muscle to bone and bone to bone, respectively. They are made up of 70% water and 30% dry mass. In total, 60–80% of this dry mass consists of collagen type I, with 2% elastin [121] that provides the tensile strength needed for movement and stability. Cartilage is a connective tissue found in joints with 60% dry mass, including 90–95% of collagen type II fibril. Collagen fibers in cartilage are arranged in a random pattern, providing tensile strength and allowing the tissue to withstand compressive forces, with a minor role of other types of collagen such as I, IV, V, VI, IX, and XI Figure 5A,B [122].

#### 4.1.1. Functions of Collagen in the Wound Healing Process

Collagen is an important component of tissues because it provides mechanical strength and elasticity side by side, acting as a natural substrate for cellular adhesion, differentiation, and proliferation. When biofilms regulate collagen through the upregulation of MMP-2, it can cause a collagen breakdown in a wound, leading to a decreased collagen I/collagen III ratio and potentially weaker repaired skin that is more prone to wound recurrence [123]. However, research has suggested that in normal, healthy tissue, the structure of collagen allows it to expose binding sites for cells and other substances that can facilitate the healing process following an injury [124]. Several recent reviews detail the roles of collagen in the skin and wounds, as mentioned in Figure 5C.

##### Role in Inflammation

Hemostasis is the first phase of wound healing and involves the body’s mechanism for stopping bleeding. During this phase, the body’s clotting mechanism is activated to stop bleeding due to the presence of collagen after exposure, leading to a fibrin clot. Inflammation is a necessary step of the healing process, as it promotes the proliferation of fibroblasts which produce collagen and other substances that help repair the damage. This inflammation process could be initiated by Collagen I and IV fragments [125]. These fragments act as a potent chemoattractant for neutrophils, enhancing immune responses and modulating gene expression [126,127]. However, it is also important for inflammation to be resolved in a timely manner to allow healing to continue. Several studies have shown that collagen plays a role in regulating inflammation and promoting the development of new blood vessels during the healing process, potentially through the action of microRNA signaling pathways [128,129].

##### Role in Angiogenesis

Angiogenesis, or the growth of new blood vessels, is a crucial process in both the normal development of the body and in the healing of wounds. It is also involved in certain diseases, such as cancer. The development and regulation of new blood vessels are carefully controlled by factors that stimulate or inhibit angiogenesis. The extracellular matrix (ECM) remodeling, which supports cells, tissues, and organs, plays a crucial role in forming new blood vessels [128,130,131,132,133]. Collagen, a backbone of the ECM, can either promote or inhibit angiogenesis depending on the specific type of collagen. Collagen type I has been shown to stimulate angiogenesis through its interaction with certain receptors on endothelial cells, especially the C-propeptide fragment, while proteolytic fragments of collagen IV and XVIII, such as endostatin and arresten, have anti-angiogenic properties and can inhibit the proliferation and migration of endothelial cells and even induce their apoptosis [134,135,136]. These findings have led to the exploration of using collagen fragments as potential therapies for inhibiting unwanted angiogenesis in various diseases.

##### Role in ECM Remodeling

Collagens are a structural component of the ECM that contribute to skin flexibility, stabilize growth factors, and regulate cell adhesion and signaling between cells and ECM. In wound healing, as the wound tissue undergoes remodeling over the years, the adult wound heals with a “normal” scar formation. The scar tissue regains anywhere from 50 to 80% of the original tensile strength of normal skin but may be functionally deficient [137]. The main difference between the scar and unwounded skin appears to be the density, fiber size, and orientation of the collagen fibrils [138].

Abnormalities in the ECM reconstitution during wound healing result in hypertrophic and keloid scars. Scarring results from altered levels of the same molecules that typically make up the ECM, i.e., collagen I and III, fibronectin, and laminin are abnormally high in scar tissue [137]. Collagen fiber orientation in scars (normotrophic, hypertrophic, and keloid) are parallel to the epithelial surface, unlike in normal skin; here, the fibers form a three-dimensional basketweave-like network [139]. There are structural and compositional differences between these types of scars. Keloid scars are characterized by abnormally thick bundles of poorly organized collagen, with fewer cross-links found in the deep dermis compared to the superficial dermis. Hypertrophic scars have thinner collagen bundles than keloid or normotrophic scars [140,141,142]. The collagen-I-to-III ratio is higher in keloids than in normotrophic scars. Even within the keloid scar, there is a heterogeneity to the collagen I/III ratio [143].

## 5. Polysaccharide-Based Biomaterials

Polysaccharides are biopolymers used as a sustainable source for multifunctional biomaterials for skin burn treatment. Natural polymers are often preferred due to their non-toxic, biocompatible, and biodegradable properties as well as their ability to be fabricated in various formulations such as hydrogels, sponges, films, nanofibers, foams, topicals, as well as wafers [144]. Unlike synthetic polymers, natural polymers are derived from biological sources such as plants or microbials and are less hydrophobic and mechanically weaker compared to synthetic-based products; therefore, their biodegradation rates are faster [145]. However, biomaterials applied on the body should not be intended to remain long-term for tissue regeneration, as they will physically impair tissue regeneration. A balance of mechanical strength and biodegradation rate characterizations is important for a successful treatment. In order to improve the mechanical strength and biodegradation rates, natural polymers are generally fabricated in hydrogels prepared by chemical cross-linking. Polysaccharide-based formulations for healing wounds have been prepared using alginate, cellulose, chitosan, and hyaluronic acid with and without synthetic polymeric mixtures [146]. This review focuses on cellulose and chitosan, which have been utilized to create a multifunctional biomaterial for skin burn wound management.

Cellulose, the main structural component of the cell walls of plants, is a linear organic polymer made up of β-1,4 combined d-glucose units linked to produce repeating parts of cellobiose. The conformation of β-linked glucose residues minimizes its flexibility because of the stabilization of the chair structure. However, the cohesive, hydrogen-bonded structure gives cellulose fibers outstanding strength and is still insoluble from water despite their high hydrophilicity [147]. Cellulose can also be sourced from bacteria such as *Acetobacter xylinum,* with attractive properties such as high water absorption, high crystallinity, a fibrous network, and high tensile strength [148].

On the other hand, chitosan is a cationic linear copolymer isolated from chitin, a key part of the crustacean exoskeleton such as crab, lobsters, shellfish, and shrimp. The beneficial functionalities of chitosan such as non-toxicity, biocompatibility and adhesiveness, biodegradability, non-antigenicity, antimicrobial properties, hemostatic effects, inertness, and wound healing properties are well documented over the last 200 years [149]. Chitosan was originally discovered by a French chemist and pharmacist, Henri Braconnot, in 1811 after an observation that chitin found in mushrooms did not dissolve in sulfuric acid [150]. 

**Figure 6:**
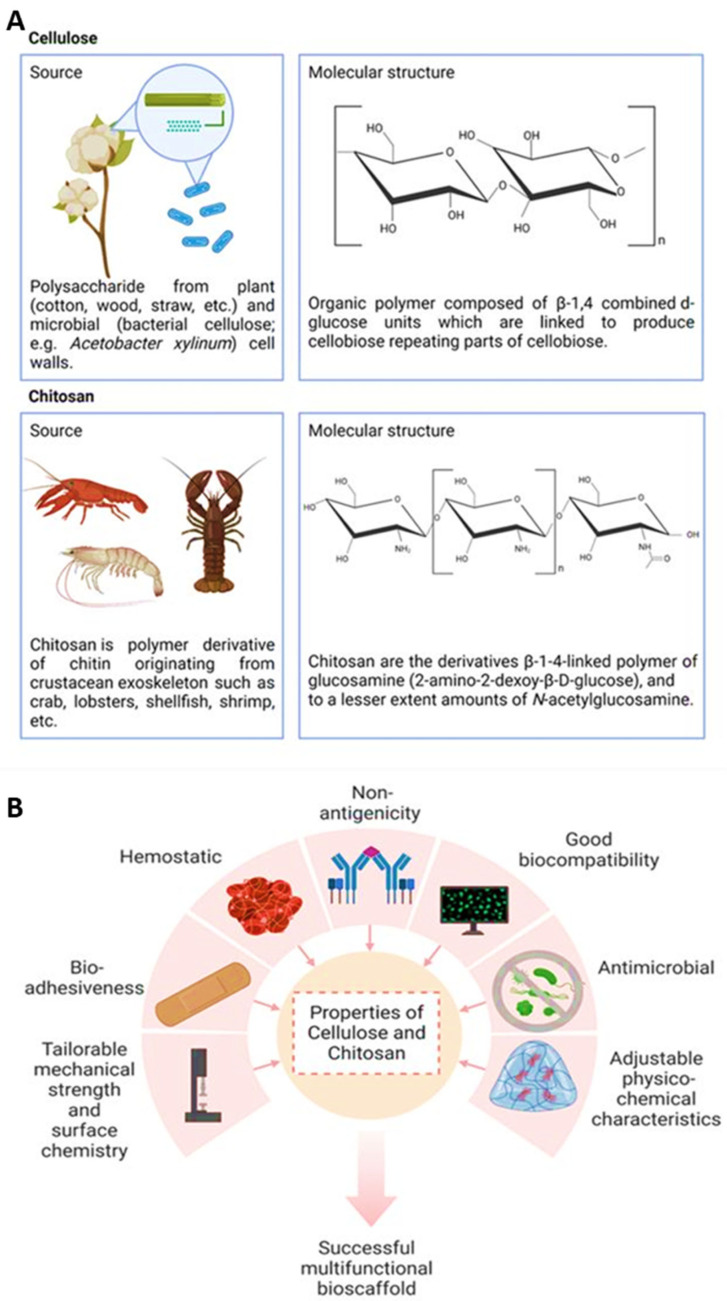
(**A**) This figure shows the sustainable sources and the chemical structural formula of both cellulose and chitosan in nature, (**B**) Different properties for cellulose and chitosan and their application in the fabrication of multifunctional bioscaffolds.

In addition, the versatility of chitosan is also due to the diverse functionalized derivatives from the chemical modification of hydroxyl and amino groups. Chitin comprises poly-*N*-acetylglucosamine, and chitosan comprises the β-1-4-linked polymer of glucosamine (2-amino-2-dexoy-β-d-glucose) and, to a lesser extent, amounts of *N*-acetylglucosamine. Other chitosan derivatives include N,O-(carboxymethyl), *N*-carboxymethyl, *N*-succinyl, *N*-acyl, *N*-carboxybutyl, *N*-carboxyethyl, 5-,methylpyrrolidinone, *N*-*N*-dicarboxymethyl, *O*-succinyl, and *O*-carboxymethyl chitosan derivatives, among others [144]. The sources and the repeated structural formula of cellulose and chitosan are illustrated in Figure 6A.

### Functions of Cellulose and Chitosan

Both cellulose and chitosan have multiple characteristics that are useful for biomaterials in tissue engineering and regenerative medicine (Figure 6B). In addition, they are sustainable, accessible, and affordable. Bacterial cellulose has been reported to be used for artificial skin, blood vessel research, and cartilage tissue engineering because of its moldability in situ whilst maintaining strength in a wet state without compromising biocompatibility [151]. Furthermore, bacterial cellulose does not undergo resorption because human cells cannot produce cellulose enzymes or cellulases [152]. Cellulose acetate scaffolds promoted the growth of functional cardiac cells in constructs [153], and TEMPO-oxidized cellulose nanofiber (TOCNF) displayed superior in vitro and in vivo inflammatory and biocompatibility compared to cellulose nanocrystals and nanofiber in rat models [154]. Modifications of microcrystalline cellulose, nanofibrillated cellulose, and nanocrystalline cellulose improved antimicrobial and wound healing applications [155].

Conversely, chitosan is also a well-known antimicrobial agent for preventing and treating infections. Its molecular weight, its degrees of deacetylation (DDA), the ionic strength and pH of the dissolving medium, and its multiple forms (films, coats, solution, hydrogel, scaffold, or combination with other materials) are all parameters that can influence antimicrobial properties [149]. The positively charged chitosan molecules and negatively charged microbial cell interaction may disrupt the microbial membrane [156]. Chitosan interaction with bacterial cell membranes may also alter cell permeability, leading to disruption [157]. Studies of chitosan on various animal models for treating or preventing wound infections show that chitosan was effective in rapid action against microbial cells in wounds and reduced mortality in cases of fatal infections [149]. Chitosan promotes granulation and organization in wound healing by enhancing the functions of polymorphonuclear leukocytes, macrophages, and fibroblasts while reducing inflammatory cells and remaining nontoxic to normal cells [158]. However, some chitosan preparations were also reported to have side effects and ineffectiveness in corneal wound healing [159,160]. Nevertheless, the benefits seem to outweigh any disadvantages. Clinical studies using chitosan to treat chronic periodontitis also reported significant improvement in the clinical parameters [161]. Chitosan can be modified with ECM components or growth factors to further increase cell adhesion, proliferation, and differentiation by modulating cellular responses. As such, different types of growth factors including basic fibroblast growth factor (bFGF), transforming growth factor-beta 1 (TGF-β1), platelet-derived growth factor-BB (PDGF-BB), and epidermal growth factor (EGF) chitosan-based bioscaffolds are used for skin, cartilage, and bone [162,163]. Chitosan can also be made into nanoparticles that have potential for healing effects on their own or as drug delivery vehicles encapsulating healing agents [164]. Combining both cellulose and chitosan allows for greater flexibility in modifying parameters to achieve ultimate effectiveness in targeted applications. Some cellulose- and chitosan-based formulated biomaterials are undergoing clinical trials or are commercial products (e.g., cellulose-based Nanoderm™ Ag and chitosan-based Axiostat^®^), which were found to be effective for the management of different types of wounds [144]. The various forms of research regarding cellulose- and chitosan-based scaffolds for skin tissue engineering are summarized in Table 2.

## 6. Sustainable Green Additives-Embedded Biomaterials

Tissue engineering is a rapidly advancing field that aims to repair and replace damaged or diseased tissues and organs. One promising area of research in tissue engineering is the use of biomaterials as scaffolds for cell growth and differentiation. To enhance the properties of these biomaterials, various additives can be incorporated. One such additive is flavonoids, which have been shown to improve the mechanical properties, biocompatibility, and antimicrobial properties of biomaterials. Other examples of additives that can be used in biomaterials for tissue engineering include growth factors, biopolymers, nanoparticles, antimicrobial agents, dyes and pigments, and ceramics and metals.

The selection of the additive and its concentration depends on the specific application and desired properties of the biomaterial. Additionally, the optimization of the final material’s properties is generally carried out through a process of trial and error, where different additives and concentrations are tested and evaluated to achieve the optimal properties for the specific application. This process can involve testing the material’s physical, chemical, and biological properties to ensure that it meets the requirements for the intended use.

### 6.1. Flavonoids

**Figure 7:**
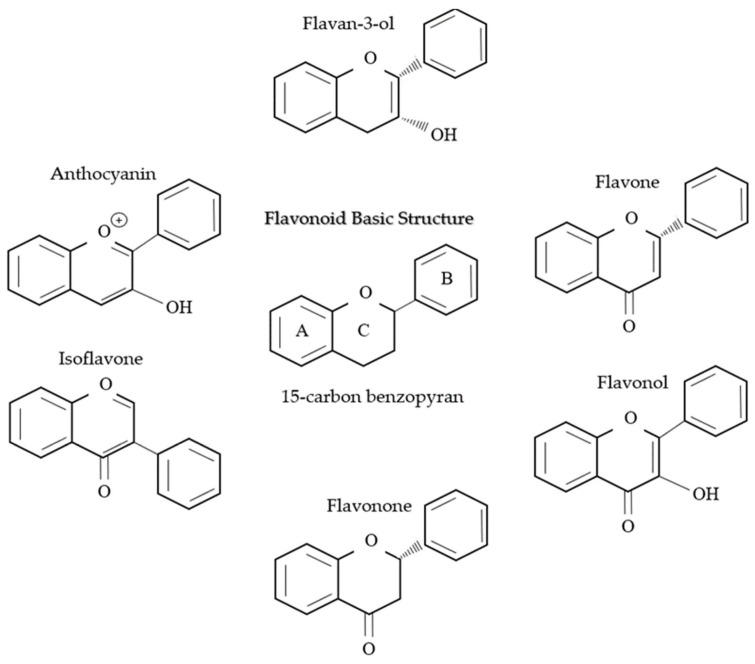
This figure shows the structure of flavonoids, which are a class of naturally occurring compounds that are widely distributed in the plant kingdom and its six subclasses. They are characterized by their distinctive chemical structure.

Flavonoids are found abundantly in plants, mainly in fruits (berries, apples, citrus foods, and grapes), vegetables (cruciferous vegetables, onion, and garlic), whole grains, teas (black and green tea), as well as red wine (great source of flavonoid), which are listed specifically in Table 3. They are a type of plant pigment or phytochemical which exert a number of health benefits and are divided into six subclasses: Anthocyanin, flavan-3-ol, flavone, flavonol, flavonone, and isoflavone. Each subclass is uniquely different in its chemical structure as well as its health benefits [173]. All the subclasses are based on the basic structure of 15-carbon benzopyran, where the phenyl group carbon bridges are cyclized with oxygen to form a flan nucleus, which is shown in Figure 7. Predominantly, flavonoids are present as 3-o-glycosides in abundance with sugar conjugates. The polymerization of flavonoids is carried out via enzymatic oxidation and fermentation; they also act as potent antioxidants and act as protection from reactive oxygen species (ROS) and inflammation in the body.

#### 6.1.1. Function of Flavonoid

Flavonoids are widely exploited for their numerous health benefits; these phytoconstituents are commonly known for their anti-oxidant and anti-inflammation properties. However, there are a number of studies that highlight the anti-cancer, hepatoprotective, neuroprotective, and anti-viral properties of these phytoconstituents. Hence, here, we discuss those properties to go through their possible mechanism on each potential property.

##### Hepatoprotective Properties

The hepatoprotective function of the phytoconstituent was tested via CCl_4_ hepatotoxicity in numerous studies; the treatment of flavonoids consistent with hepatoprotective results was demonstrated with the reduction in or restoration of glutathione levels in the liver, the elevation of total protein serum, normal activities of SOD and catalase, and the impairment of CYP2E1 expression [176,177,178].

##### Anti-Viral Properties

Numerous published articles have scientifically proven the anti-viral properties of flavonoids [179]. In a study, Wang et al. tested flavonoid compositions towards the enterovirus and showed anti-viral properties by inhibiting the viral replication as well as inhibiting the viral protein synthesis. Dell’Aica et al. studied the anti-viral properties of flavonoids against HIV I, which show positive results against the virus by inhibiting the replication and infection capacity of HIV in an in vitro model. A different study showed flavonoids’ antagonistic properties against the virus reverse transcriptase by forming chemokines complexes and hence progressively diminishing the HIV I bind receptor’s ability [180].

##### Anti-Bacterial Properties

Flavonoids are also well known for their anti-bacterial properties by neutralizing bacterial toxin [179]. A study conducted by Dell’Aica et al. proves EGCG has antibacterial properties against *Ba. Anthracis* by neutralizing the bacterial toxin as well as inhibiting the energy metabolism and nucleic acid synthesis [180].

##### Anti-Cancer Properties

The anti-cancer properties of flavonoids may work via different mechanisms; a study conducted by Arif et al. discovered that the anti-cancer properties of flavonoids work by the breakage of cellular DNA via flavonoids-induced redox cycling, which leads to pro-oxidant cancer cell death [181]. Another study stated the phytochemical fights against cancer through enhancing cells apoptosis as well as immunity activation and the inhibition of tumor angiogenesis activity.

##### Neuroprotective

The pathophysiology of neuro-related disease is connected to oxidative stress. When the dynamic balance of ROS hemostasis is disrupted, the elevation of ROS in the hippocampus might happen, hence leading to impaired brain function. In an Alzheimer’s model, Yang et al. studied the neuroprotective activity of flavonoids and discovered that these phytoconstituents protect the brain by the up-regulation of the MAPK pathway, inducing the apoptosis factor, the up-regulation of Bcl-2, and the inhibition of NLRP3 activation [176].

##### Anti-Inflammation

Numerous studies demonstrated the anti-inflammatory mechanism of flavonoids through the reduction in LPS-induced tumor necrosis factor-α (TNF-α) and interleukin-1 (IL-1) as well as the elevation of macrophages [176]. Zhu et al. demonstrated the anti-inflammation properties exerted by flavonoids through the inhibition of LPS-induced apoptosis as well as the inhibition of lipid anabolism gene expression. They also validated the role of flavonoids in diminishing inflammation via reducing the pro-inflammatory cytokines and hence elevating anti-inflammatory cytokines, which includes the inhibition of nuclear transcription factors [182]. The therapeutic applications and and mechanism have been stipulated in Table 4.

## 7. Flavonoid-Incorporated Biomaterial

Multifunctional biomaterials are well exploited nowadays due to their multiple action mechanisms, which can be manipulated according to the desired function. This review mainly focuses on Quercetin, EGCG, and kaempferol incorporated in biomaterial for skin burns or chronic skin wounds.

### 7.1. Quercetin-Embedded Biomaterial

Quercetin is a flavonoid that is widely known for its antioxidant effects and is naturally found in plants, such as apples, onions, and berries. This potent flavonoid has the ability to aid wound healing by diminishing inflammation and oxidative stress, and it has the potential to promote collagen production and cell growth and mitigate the pro-inflammatory cytokines released [177]. It has also been proven that quercetin stimulates dermal cells, enhancing collagen formation and tissue repair [178]. Furthermore, a number of studies have found angiogenic and anti-bacterial properties, and it protects against oxidative damage to aid in cell proliferation.

A study conducted by Ajmal et al. showed the antibacterial wound healing activity exerted by PLC-gelatin and quercetin nanofiber. The disk diffusion assay against *S. aureus* showed that quercetin exerts antibacterial properties by zone inhibition. The in vivo activity was observed in rat models with a full-thickness skin wound, where the quercetin-embedded nanofiber was 100% healed within 16 days of treatment. The histology results showed an abundance of collagen synthesis. Their previous study showed that the nanofiber exerts multiple mechanisms such as free radical scavenging and protection against oxidative damage and lipid peroxidation, aiding in fibroblast proliferation [179].

Vedakumari et al. created a novel quercetin-embedded chitosan fibrin scaffold to accelerate wound healing, acting as wound dressing for future applications. The quercetin sponge obtained the fastest complete epithelialization at day 16, whereas all the other groups obtain 100% wound closure up to 29 days. The in vivo study suggested that the quercetin-embedded sponge significantly expedited the wound healing process, successfully creating a multifunctional bioscaffold [180].

### 7.2. EGCG-Embedded Biomaterial

Epigallocatechin gallate (EGCG) is the most abundant polyphenolic compound present in *Camelia sinensis*, widely known as green tea; it is a potent antioxidant and free radical scavenger that is vital in the treatment of burns or chronic wound healing. There are also a number of mechanisms exerted by EGCG which are vital in aiding wound healing activities such as: inhibiting the transcription of nuclear factor-kappa B (NF-κB), inhibiting (NF-κB) protein factors, inhibiting the production of IL-8, inhibiting inflammation induced by LPS, as well as SOD activation [180].

In a study, Kim et al. designed an EGCG-embedded collagen sponge which successfully enhances the wound healing of diabetic mice via rapid epithelialization, angiogenesis, and the reorganization of tissue granulation by activating the myofibroblast. The sponge was incorporated with 10, 100, and 1000 ppm EGCG, where 10 ppm EGCG shows the fastest wound healing at day 14 compared to all the other groups. The 10 ppm EGCG-embedded group also showed faster re-epithelialization as well as angiogenic activity in the immunohistochemical analysis [182]. Their aim was to create a chronic wound healing supportive agent. The various forms of research regarding flavonoids incorporated scaffolds for skin tissue engineering are summarized in Table 5.

Sun and his colleagues created a bioinert EGCG-embedded nanoparticle to combat chronic wounds. On day 10, the treated wound showed a completely healed epithelium in the quercetin treatment group with visible hair follicles and the neovascularization of the vessel, with a significantly higher healing rate compared to that of other groups (*p* > 0.001). The in vivo study suggests that the nanoparticles aid in accelerating collagen accumulation and rapid angiogenesis and mitigate the inflammation cells infiltration on the wound side, hence corelating with their objective of creating a biomaterial for aiding chronic wound healing [186].

Zhao et al. created a novel self-healing multifunctional hydrogel where the EGCG-embedded hydrogel showed a higher and significant wound healing closure compared to the commercialized Tegaderm product. Other than that, the hydrogel also shows good anti-oxidant properties via the ROS assay and hence might be the cause of rapid wound healing in the in vivo study [183].

### 7.3. Kaempferol-Embedded Biomaterial

Kaempferol, a potent flavonoid classified as a flavanol, has comparable wound healing properties to quercetin. Not only that, this flavanol is also widely studied for its hepatoprotective effect, whereas in wound healing, the incorporation of kaempferol was shown to increase the proliferation of dermal cells and litigate inflammation [186]. Zeka et al. tested kaempferol-embedded hydrogel on a skin model to test the biocompatibility towards the cell culture. They targeted this study towards ulcers, abrasions, and burn wounds; the study showed that the hydrogel successfully aided in the rapid cell proliferation of the skin model [185]. This shows good biocompatibility; hence, it can be used in further studies for wound healing activities.

## 8. Conclusions

In conclusion, burns are a significant global public health issue affecting many people worldwide. The treatment of burns has traditionally been focused on controlling pain, removing dead tissue, preventing infection, reducing scarring risk, and promoting tissue regeneration. However, these traditional methods are often associated with negative environmental impacts and poor biocompatibility. Tissue engineering and the use of sustainable biomaterials have emerged as promising alternative treatment options for burns. Green biomaterials such as collagen, cellulose, and chitosan effectively promote wound healing while reducing the risk of infection and minimizing scarring and tissue damage. These materials are biocompatible, biodegradable, environmentally friendly, and cost-effective.

The use of multifunctional green biomaterials can revolutionize how we treat skin burns, promoting faster and more efficient healing. These materials have been found to have other benefits such as reducing inflammation and promoting angiogenesis. Using these sustainable biomaterials can also help in reducing the environmental impact of their production and disposal. In light of the many benefits of these sustainable biomaterials, further research needs to be conducted to explore their full potential in the treatment of burns. In conclusion, the use of green biomaterials in burn wound treatment is a promising avenue of research that has the potential to improve the lives of burn victims worldwide greatly.

## Figures and Tables

**Table 1:** The current commercialized skin substitutes.

Skin Substitutes	Structure	Example of Skin Substitutes
Dermo-epidermal	Bilayered biomaterial; mimic both the dermis and epidermis	Apligraf^®^ [89,90,91] OrCel^®^ [92], Biobrane^®^ [93,94], Hyalomatrix^®^ [95,96] Pelnac^®^ [97,98], TransCyte^®^, StrataGraft^®^ [99]
Dermal	Biomaterial-based matrix; mimic the skin dermis	AlloDerm^®^ [100], Dermagraft^®^ [101], Integra^®^ [102,103,104], and MatriDerm^®^ [105], Insuregraf^®^ [106], Nevelia^®^ [107], Primatrix™ [108]
Epidermal	Thin biomaterial-based membranes; mimic the epidermis	Cultured epithelial autograft: Epicel^®^ [109], EpiDex^®^ [110], JACE^®^ [111] Suspended cultured autologous cell: ReCell^®^

**Table 2:** The different forms of cellulose- and chitosan-based scaffolds for skin tissue engineering.

Biomaterial Type	Composition	Application	Outcomes	References
Smart hydrogels	Chitosan, glycerol phosphate sodium, and cellulose nanocrystals with encapsulated human umbilical cord–mesenchymal stem cells (hUCMSCs)	Full-thickness cutaneous skin wound	Low toxicity; accelerated wound closure; re-epithelialization; anti-inflammatory	[165]
Smart hydrogels	Chitosan, TEMPO-oxidized cellulose nanofiber (TOCNF), and β-glycerophosphate (hermos-sensitive)	Skin burn	TOCNF integration improved the acute response with prominent M2 macrophage cells	[166]
Smart hydrogels	Chitosan, hydroxypropyl methylcellulose (HPMC), and glycerol (thermo-sensitive)	Skin wound	Biodegradable, controlled release, and low cytotoxicity	[167]
Self-healing hydrogels	Carboxymethyl chitosan (CMC) and dialdehyde-modified cellulose nanocrystal (DACNC)	Deep partial-thickness burned wound	Superior biocompatibility; scar formation suppression	[168]
Film	Quaternized hemicelluloses (QH) and chitosan with epichlorohydrin (ECH) as a crosslinker.	Skin wound	Drug-loading capability, controlled release, and biocompatible	[169]
Drug-loaded nanocomposites	Copper (CU) nanoparticles (NPs)-loaded chitosan-attached cellulose fibers	Skin burns and wound dressing	Ion release, biocidal action against *E. coli*	[170]
Electrospun nanocomposites	Fiber mats of a chitosan–polyethylene oxide matrix reinforced with cellulose nanocrystals (CNCs)	Skin wound	Non-cytotoxic impact on adipose-derived stem cells (ASCs)	[171]
Sponge	Chitosan and cellulose composites; recyclable method	Skin wound	Anticoagulated whole blood absorption, antimicrobial, anti-inflammatory, biocompatibility with human fibroblasts.	[172]

**Table 3:** The subclasses of flavonoids, their composition, and natural resources.

Flavonoid Subclass	Compositions	Natural Sources	Ref.
Anthocyanidins	Cyanidin Peonidin Pelargonidin Malvidin Delphinidin	Berries: red, blue, purple Grapes: red wine, purple	[174,175]
Flavonols	Kaempferol Quercetin Myricetin Isorhamnetin	Kale Broccoli Apples Berries Teas	[174,175]
Flavan-3-ol	Monomers: Epigallocatechin Catechin Epicatechin Polymers: Proanthocyanins	Apples Berries Teas Grapes Chocolate	[174,175]
Flavanone	Hesperetin Naringenin Eriodyctiol	Citrus fruits: orange, grapefruit, lemon	[174]
Flavone	Apigenin Luteolin	Parsley Thyme Celery	[174]
Iso-flavone	Genistein Glycitein Daidzein	Soy: foods, beans Legumes	[174]

**Table 4:** Flavonoids’ therapeutic applications and their potential mechanism.

Flavonoids’ Functionality	Mechanism	Ref.
Hepatoprotective	Restores glutathione levels Maintains SOD and catalase activity	[183]
Anti-inflammatory	Reduction in lipopolysaccharide (LPS) induced: tumor necrosis factor-α (TNF-α), interleukin-1 (IL-1) Macrophages elevation TNF- α suppression	[184,185]
Anti-bacterial	Cytoplasmic membrane impairment Inhibition of energy metabolism Inhibition of nucleic acid synthesis	[183,186]
Anti-viral	Inhibition of viral protein synthesis Inhibition of viral replication	[183,186]
Anti-cancer	Cell DNA breakage by copper ions Enhanced cells apoptosis	[183,175]
Neuroprotective	Regulates MAPK Regulates Bcl-2 Inhibits the activation of NLRP3	[184]

**Table 5:** Flavonoids incorporated in different types of bioscaffolds and their effect.

Biomaterial	Composition	Study Design	Result	References
Sponge	EGCG	In vivo Immunohistochemical analysis	In vivo: (Day 14) 10 ppm EGCG shows the fastest wound recovery. IHC: (10 ppm EGCG) Rapid keratinocytes proliferation, (Ki-67) promotes re-epithelialization, (α-SMA) induces myofibroblast, (CD31) blood vessel formation.	[182]
	Quercetin	In vivo	In vivo: (Day 16) 100% wound closure	[180]
Hydrogel	Quercetin	In vivo	In vivo: Significant wound closure (%), rapid collagen deposition	
	EGCG	ROS analysis In vivo	ROS assay: Reduction in intracellular ROS Wound: (Day 18) 2.0% significance compared to the commercialized product and thicker tissue granulation	[183]
	Kaempferol	Skin model	Skin model (mice fibroblast): Rapid cell proliferation	[184]
Nanofiber	Quercetin	Antioxidant assay Antibacterial study In vivo	Antioxidant assay: DPPH >50% scavenged Antibacterial study: exerts antibacterial properties by the zone of inhibition In vivo: (Day 16) 100% wound closure	[180]
Nanoparticles	EGCG	In vivo Immunohistochemical analysis	In vivo: (Day 10) 100% wound closure IHC: (EGCG group) Formation of blood vessels, hair follicles, an epithelium, and a thinner epidermis	[186]

## Data Availability

The data presented in this study are available on request from the corresponding author.
